# Nystagmus in infancy: causes, characteristics and main tools for diagnosis

**DOI:** 10.1038/s41433-024-03504-4

**Published:** 2024-12-05

**Authors:** Emanuel Goldman, Karen Hendler, Claudia Yahalom

**Affiliations:** 1 Faculty of Medicine, Hebrew University of Jerusalem, Jerusalem, Israel; 2 Department of Ophthalmology, Hadassah Medical Center, Jerusalem, Israel

**Keywords:** Eye diseases, Paediatrics

## Abstract

**Background:**

Nystagmus in infancy may occur as part of an ocular condition, a neurological disorder or be idiopathic. The objective of our study was to evaluate the main causes for nystagmus in infants aged 0–1 year, the accompanying clinical characteristics and the effectiveness of different diagnostic tests used for reaching definite diagnosis.

**Methods:**

A retrospective study was conducted on medical records of infants who were seen at a tertiary hospital between 2016 and 2021. Clinical data were obtained including age at presentation, presence of strabismus, refractive errors, auxiliary tests performed to reach diagnosis, and initial versus final diagnosis.

**Results:**

This study included 147 infants. The most common ocular pathology causing nystagmus was albinism followed by inherited retinal diseases (IRDs). Main tests that helped reach diagnosis included clinical examination, genetic testing and electroretinogram (ERG). Vertical nystagmus was seen in 8/147 infants; among them 4 had an IRD as a final diagnosis. The most common associated clinical characteristics were high hypermetropia and strabismus.

**Conclusions:**

The most common causes for nystagmus during the first year of age were albinism and IRDs. The most useful diagnostic tests to reach diagnosis were ERG together with genetic testing for IRDs and clinical ophthalmologic examination for albinism. IRDs play an important role as the cause for nystagmus in infants; this diagnosis can be often missed during the first months of life and a vertical nystagmus wave is commonly observed in this group. Nystagmus secondary to a neurologic condition is rare in this age group.

## Introduction

Nystagmus is a rhythmic and involuntary movement of either one or both eyes, involving two phases leading the eyes to move away from and then return to the target. It might occur in the horizontal, vertical or torsional plane and can be further classified into a jerk or pendular waveforms [1]. The pathophysiology behind the appearance of nystagmus, especially in infants, has still many unanswered questions, with several possible hypotheses published in the literature [2].

The prevalence of nystagmus in children ranges from 14 per 10,000 to 6.1 per 10,000 live births with an increased prevalence of children born prematurely [3, 4].

Nystagmus in children can be roughly divided into two main groups [5]: “Infantile nystagmus syndrome” (INS) when the nystagmus appears for the first time before age 6 months, and “Acquired nystagmus” in which appearance of nystagmus occurs later in life. Idiopathic nystagmus is a term used when all known causes for nystagmus have been ruled out [5].

The most common forms of INS are nystagmus associated with albinism or retinal diseases and idiopathic nystagmus [6]. In nystagmus associated with other eye diseases, vision is not only affected by the excessive motion of the image on the retina, but also by a defective visual system [1]. Based on the fact that in many cases the retina itself may, at least in-part, cause reduced visual acuity, a research group in Leicester have used optical coherence tomography (OCT) to explore the relationship between retinal morphology and vision, and develop the Leicester OCT grading system, that helps to provide a prognostic indicator for vision [7, 8].

Several genes have been identified to cause nystagmus. Recently, the FRMD7 gene was pinpointed as a primary contributor to hereditary nystagmus [9]. The main aetiologies for acquired nystagmus are related to neurological pathology [10].

Clinical descriptions of nystagmus are based on the direction of the fast phase and according to the waveform that the eye movement creates. Published literature shows that horizontal waveform is generally associated with INS, and a vertical waveform is commonly associated with nystagmus of acquired or neurological origin, but it is not possible to conclude what the aetiology is unequivocally based on the waveform [11].

Management of children with nystagmus varies widely and includes anamnesis, an orthoptic examination, refraction, complete eye examination, electrophysiology, OCT, genetic testing and magnetic resonance imaging (MRI) in some cases [6, 12].

The primary objective of our study is to study population-based estimations concerning the primary causes and the clinical characteristics of nystagmus in infants up to one year of age. We also aimed to assess the sequence and timing of diagnostic tests and their yield in reaching an ultimate diagnosis.

## Methods

A retrospective study of 147 infants referred due to nystagmus to our clinic was carried out in a national referral centre for children with low vision located at a tertiary hospital. Medical charts of infants up to the age of one year that underwent eye examination between the years 2016–2021 were reviewed; those without nystagmus at the initial examination or without further follow up visits were excluded from the study. Data were collected from medical records of studied patients and included results of ophthalmologic exam, ancillary tests such as ERG, visual evoked potentials (VEP), MRI, and genetic testing when available. We have recorded information regarding all the tests performed and in which order for each patient, as well as the main test that led to the definite diagnosis. Two sub-groups were created for further analysis: infants younger than 6 months of age and infants aged 6 months and older. Ethical approval for this study, according to the Helsinki tenets, was obtained from the hospital Institutional Review Board.

Statistical analyses were performed using the Statistical Package for the Social Sciences software version 27 (SPSS Inc., Chicago, IL, USA). P value of <0.05 was considered statistically significant. Examination of the relationship between two qualitative variables was performed by applying Chi-square and Fisher’s exact test. Comparing a quantitative variable between 2 groups, was performed by applying the *t* test, and for the purpose of comparing a quantitative variable (age of diagnosis) between 3 or more groups, the repeated measures Analysis of Variance model (ANOVA model) was applied.

## Results

### Baseline demographics

Our cohort included 147 infants with nystagmus, of whom 83 were male (56.5%) and 64 were female (43.5%). At their first visit, 84 (57.2%) of the studied infants were under 6-month-old and 63 (42.8%) of them were 6 months old or older.

### Suspected diagnoses at first examination compared to final diagnosis

**Fig. 1:**
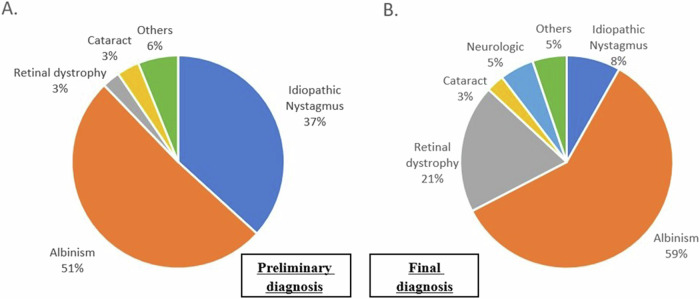
Diagnosis at first examination versus final diagnosis. Prevalence of nystagmus etiologies among all studied infants (*n*=147). **A** At first examination and **B** final diagnosis; Others include Aniridia, Coloboma and other rare diagnosis.

The 2 most common diagnoses at first examination were albinism (*n* = 75, 51.0%) and idiopathic nystagmus (*n* = 54, 36.7%) followed by cataract (*n* = 5, 3.4%), IRDs (*n* = 4, 2.7%) and others (*n* = 9, 6.1%) (Fig. 1).

The confirmed final diagnoses indicated ocular pathology in 140 patients, with albinism being the most common diagnosis in 87 cases (59.2%) (Chi square test *p* < 0.001), IRDs in 28 cases (19.5%), idiopathic nystagmus was observed in 12 cases (8.2%), while cataract was present in 4 cases (2.7%) and 9 (6.1%) patients were diagnosed with other ocular pathologies. Additionally, neurological causes were identified in 7 (4.7%) patients (cerebral visual impairment (CVI, *n* = 4), space-occupying lesion (SOL, *n* = 1), moebius syndrome (*n* = 1), transient hypomyelination (n = 1)) (Fig. 1).

**Fig. 2:**
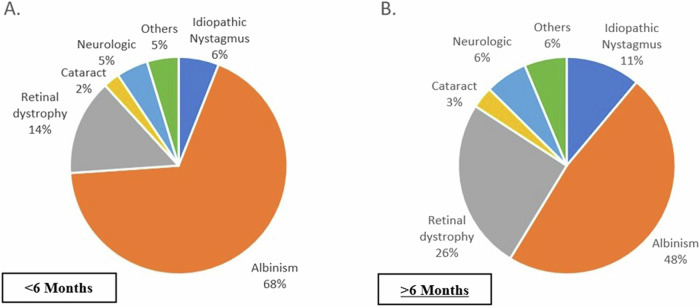
Final diagnosis prevalence between different age groups. Prevalence of nystagmus etiologies among the two-age groups. **A** <6 Months (*n*=84) and **B** ≥6 months (*n*=63). Others include Aniridia, Coloboma and other rare diagnosis.

When analysing the two age sub-groups, albinism was the most common aetiology in both groups, but significantly higher in the group under six months of age with 57 (67.9%) patients. In infants over six months of age, an increase in the incidence of IRDs can be detected (Fig. 2).

### Clinical features

Strabismus was found in 51 (34.7%) infants, mainly esotropia (42/51, 82.4%) (*p* = 0.028). Refractive errors were common, significant hypermetropia (defined as ≥4 Dioptres) was found in 73 out of 147 (49.6%) and only 9 (6.1%) had significant myopia defined as ≤ (-4) (*p* = 0.010). Significant Astigmatism of >2 dioptres reported in 50 (34%) patients, most of them diagnosed with albinism (*p* = 0.023).

The most common nystagmus waveform was horizontal (*n* = 122, 82.9%), followed by vertical nystagmus in 8 (5.4%) patients and a mix pattern in 9 (6.1%) of studied infants. Among the infants with vertical nystagmus, 4 had an IRD as a final diagnosis (achromatopsia *n* = 3, CSNB *n* = 1), the others had aniridia (*n* = 2), cataract (*n* = 1), and idiopathic infantile nystagmus (*n* = 1).

### Diagnostic workup

All 147 infants underwent ocular examination by a paediatric ophthalmologist upon their first visit to our clinic, 105 (71.4%) had genetic tests and 41 (27.9%) completed electrophysiology tests including ERG and VEP.

The most common test performed as “test 1” was MRI, including infants who underwent this test prior to their first examination in our clinic. Genetic tests were the most common “test 2, 3, and 4”. ERG/VEP were the second most common “test 2 and 3” (Table 1).

**Table 1:** Diagnostic tests performed and chronological timing.

	Test 1		Test 2		Test 3		Test 4		Total
	Count- (30)	%	Count-(99)	%	Count- (52)	%	Count- (24)	%	147
MRI	21	70%	8	8.1%	0	0	0	0	29 (19.7%)
US	5	16.7%	4	4%	0	0	0	0	9 (6.1%)
Head CT	2	6.7%	1	1%	0	0	0	0	3 (2%)
Genetics	1	3.3%	63	63.6%	26	50%	15	62.5%	105 (71.4%)
ERG/VEP	1	3.3%	20	20.2%	18	34.6%	2	8.3%	41 (27.9%)

*MRI* magnetic resonance imaging, *US* ultrasound, *Head CT* head computed tomography, *Genetics* all molecular genetic testing, *ERG/VEP* electroretinogram/ visual evoked potential.

MRI was performed in 29/147 (19%) of studied infants, whereas a diagnosis of a neurologic cause for nystagmus was reached only in 6/29. The remaining patient with a neurological cause was diagnosed clinically (Moebius syndrome).

OCT was performed in 15 infants only, with relatively poor image quality.

**Fig. 3:**
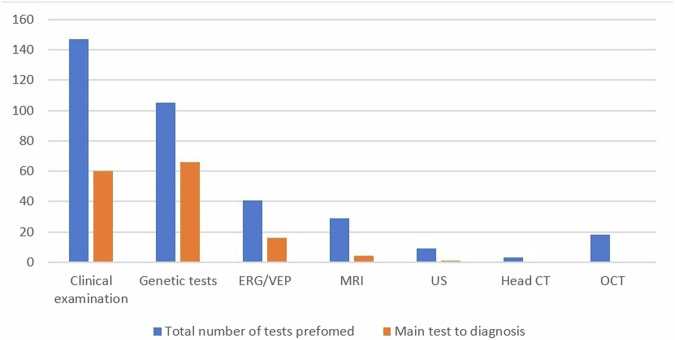
Distribution of main identified genes. *Tyr* (*n*=32); *OCA2* (*n*=11); *FRMD7* (*n*=1); *SLC38A8* (*n*=2); retinal dystrophy (*n*=13) (including *TRPM1*, *CRB1*, *CNGA3*, *CNGB3*, *ALSM1*, *GUCY2D*, *CRX*, *IQCB1*, *RPE65*); neurological (*n*=7) (including *ADRA2B*, *ITPR1*, *TMEM63A*, *ASH1L*, *CDKL5*, *CACNA1A* chromosome 1p36); *PAX6* (*n*=2).

The main tests to reach a final diagnosis were Genetic testing (*n* = 66, 44.9%), clinical examination (*n* = 60, 40.8%), and ERG (*n* = 16, 10.9%). Notably, clinical examination demonstrated significant efficacy in diagnosing albinism, representing 72% of studied infants (43 out of 60 cases) (Fig. 3).

**Fig. 4:**
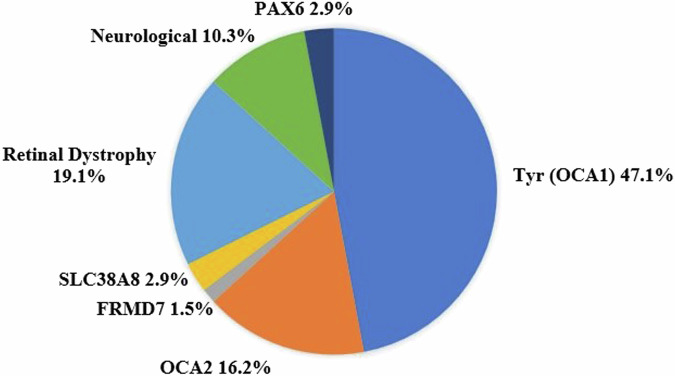
Effectiveness of diagnostic tests. Total tests performed (blue columns) in relation to the number of tests that led to final diagnosis (orange columns).

Pathogenic variants were found in 68 out of 105 patients (65%) who underwent genetic testing. Two of the genetic tests yielded only one pathogenic variant of an OCA gene, thus weren’t considered as definite diagnosis. *Tyr* (OCA1) mutation and IRDs causing genes were the most common genes identified, with 32 (47.1%) and 13 (19.1%) infants, respectively (Fig. 4).

## Discussion

The process of determining the cause of nystagmus in infants is challenging due to limited cooperation related to age, variability in nystagmus presentations as well as the multitude of potential existing aetiologies. Consequently, a comprehensive diagnostic approach entails the judicious selection of appropriate tests based on clinical characteristics [5, 13, 14].

Our findings, consistent with prior research, indicate that INS secondary to ocular pathology and idiopathic nystagmus collectively account for nearly 90% of cases, with albinism being the predominant cause in infancy, observed in over half of the cases [4, 13, 15, 16]. While albinism, most of the time, can be clinically diagnosed by an ophthalmologist without any further testing, we identified 12 (8.2%) patients whose final diagnosis shifted from a preliminary diagnosis of idiopathic nystagmus to albinism.

IRDs were found as the second most prevalent cause of INS in this age group. Among 24 infants that initially had a diagnosis of idiopathic nystagmus, final diagnose changed to IRDs after performing ERG and genetic testing. This underscores the critical role of thorough clinical evaluation by ophthalmologists and the necessity of additional testing in determining the aetiology of nystagmus, particularly in IRDs.

Regarding age of diagnosis in our cohort, the diagnosis of albinism was reached predominantly before the age of six months, as shown in previous research indicating it as the primary cause of infantile nystagmus [4, 16]. IRDs were also identified during the first six months of age as a common cause of infantile nystagmus, but there was a slight increase in the prevalence of this diagnosis beyond six months of age. Notably, existing literature lacks evidence of such an uptick in retinal dystrophy incidences within this age group. We suggest that this increase indicates that its clinical signs even though appear during the first months of life, are still subtle, thus delaying the diagnosis. Furthermore, it’s known that retinal maturity is not complete before the age of six months, which could cause delays in performing ERG tests and achieving a diagnosis [17].

Traditionally, vertical nystagmus has been predominantly associated with neurological origins and intracranial pathologies in the literature [18, 19]. However, more recent studies have demonstrated that vertical nystagmus may not exclusively imply a neurological cause but can also manifest in cases of IRDs, albinism, and idiopathic nystagmus [11, 20]. In our study, we observed that all patients diagnosed with nystagmus due to neurological cause exhibited a horizontal waveform pattern, whereas none of those with vertical nystagmus were found to have a neurological cause. Among the eight children with vertical nystagmus, IRDs accounted for the cause in 50% of them. These findings underscore the notion that vertical nystagmus patterns may suggest a neurological cause but do not exclude ocular aetiologies, particularly IRDs. Therefore, it is crucial to consider and investigate these potential aetiologies alongside neurological ones.

In this study, we found the genetic tests and ERG tests were the most common tests to reach definite diagnosis. Their high diagnostic value in our cohort, consistent with previous publications, corresponds to the accepted management protocols for nystagmus [6, 12, 13].

Neurological causes of nystagmus in infancy are exceedingly rare [10, 16]. Our data shows that only 4 infants (13.8%) who underwent an MRI as the first test, had a definite diagnosis from this test. This is in accordance with a prior study that found, that the most common first test carried out was an MRI with a diagnostic yield of only 16% [13]. Self et al proposed diagnostic workflow to guide clinical practice among paediatric nystagmus specialists, aiming to focus the diagnostic process for as many children as possible and to try to avoid performing unnecessary invasive tests such as MRI [6].

In our cohort, OCT had limited diagnostic value. We believe that the lack of a hand-held OCT device in our service, that has been proven to be particularly useful in pre-verbal infants with challenging cooperation, is the reason for the low additional diagnostic value of OCT in our studied infants [21].

Additional clinical characteristics within our cohort show a high percentage of strabismus, specially esotropia, in coincidence with previous published data [22].

The limitations of our study encompass its retrospective design limiting available data. Furthermore, our study was based on data from an ophthalmological unit, not including infants that might have arrived at an emergency unit or paediatric neurologists for consultation due to nystagmus.

In conclusion, the most common causes for nystagmus in our cohort were albinism and IRDs. The main tools to reach final diagnosis were clinical ophthalmologic examination for albinism and electrophysiology together with genetic testing for IRDs. Nystagmus secondary to neurologic conditions is rare in this age group. Vertical nystagmus is uncommon and is often associated with IRDs. Special care should be taken in infants with nystagmus during the first months of life in order not to miss the diagnosis of IRDs.

## Summary

### What was known before

*Nystagmus in infancy is relatively rare and is generally caused by and ocular (mainly retinal dystrophies) or neurological condition
*Vertical nystagmus is traditionally associated with neurological conditions The most common used test performed in infants with nystagmus is head MRI

### What this study adds

- The most common causes for nystagmus in infants are albinism and inherited retinal dystrophies
*The most efficient test to reach final diagnosis are careful clinical evaluation for albinism; and genetic testing/electrophysiology for inherited retinal diseases
* The diagnostic yield of MRI is low for this age group

## Data Availability

Full retrospective data is available upon request.
